# Analysis of the disease burden trend of malignant tumors of the female reproductive system in China from 2006 to 2020

**DOI:** 10.1186/s12905-022-02104-2

**Published:** 2022-12-07

**Authors:** Xiting Han, Zhihong Wang, Dongmei Huang, Kehong Deng, Qian Wang, Cancan Li, Jicun Zhu

**Affiliations:** 1 Department of Obstetrics and Gynecology, The Second Affiliated Hospital of Zhengzhou University, 2 Jingba Road, Zhengzhou, 450001 Henan People’s Republic of China; 2 College of Public Health, Zhengzhou University, 100 Kexue Avenue, Zhengzhou, 450001 Henan People’s Republic of China

**Keywords:** Female reproductive system, Malignant tumor, Mortality, Disease burden

## Abstract

**Background:**

Malignant tumors of reproductive system seriously threaten women’s life and health. We analyzed the changes in mortality and disease burden of cervical cancer, uterine cancer and ovarian cancer in China from 2006 to 2020 to provide a basis for formulating scientific prevention and control measures.

**Methods:**

Annual death data for cervical cancer, uterine cancer and ovarian cancer were collected from the Chinese Cause of Death Surveillance. The crude mortality rate (CMR), age-standardized mortality rate (ASMR), annual percentage change (APC), and average APC (AAPC) were applied to analyze the trend of mortality. Loss of life expectancy (LLE) and years of life lost (YLL) were used to assess disease burden.

**Results:**

From 2006 to 2020, there was no significant change in the total ASMR and standardized YLL rates of malignant tumors of the reproductive system, leading to an average LLE of 0.18 years. The YLL rate was the highest in the 55–59 age group. The mortality rate and disease burden of the three types of cancer have changed from uterine cancer higher than cervical cancer and ovarian cancer in 2006 to cervical cancer higher than ovarian cancer and uterine cancer in 2020. The ASMR and standardized YLL rate of uterine cancer showed a downward trend, and AAPC was − 5.21% (− 9.31% ~  − 0.91%) and − 6.07% (− 9.45% ~  − 2.58%), respectively. The mortality rates of cervical cancer and ovarian cancer remain high.

**Conclusion:**

The mortality and disease burden of malignant tumors of the female reproductive system in China are still at a high level. It is necessary to improve screening and prevention strategies as soon as possible, improve the techniques of diagnosis and treatment, and take adequate measures to protect women's life and health.

**Supplementary Information:**

The online version contains supplementary material available at 10.1186/s12905-022-02104-2.

## Plain English summary

Malignant tumors of the female reproductive system are the leading causes of women's mortality worldwide, mainly including cervical cancer, uterine cancer and ovarian cancer. Numerous studies have reported mortality changes in the three primary reproductive system cancers among different countries, with inconsistent temporal trends. In 2020, almost 17.51% of malignant tumors of the female reproductive system deaths occurred in China because of the large population base and severe aging. However, there are limited studies on the disease burden of malignant tumors of the female reproductive system in China. This study analyzed the mortality, YLL and LLE of cervical cancer, uterine cancer and ovarian cancer using the National Death Cause Surveillance Dataset from 2006 to 2020. The findings revealed that mortality and disease burden of malignant tumors of the reproductive system showed no significant change. The mortality and disease burden of uterine cancer decreased significantly, but that of cervical cancer and ovarian cancer remain high. Cervical cancer has a higher mortality and disease burden than uterine cancer and ovarian cancer, making it the most severe malignant tumor of the female reproductive system. Screening and HPV vaccination will reduce the mortality and disease burden of cervical cancer in China, but this effect will take many years to manifest. Screening and prevention strategies for high-risk groups of uterine cancer and ovarian cancer should be established as soon as possible. In summary, the management and monitoring of malignant tumors of the female reproductive system should be strengthened.

**Supplementary Information:**

The online version contains supplementary material available at 10.1186/s12905-022-02104-2.

## Introduction

Cervical cancer, uterine cancer and ovarian cancer are the most common malignant tumors of the female reproductive system. According to GLOBOCAN (Global Cancer Statistics) 2020, the three types of cancer have caused about 342,000, 97,000 and 207,000 deaths worldwide, respectively, seriously threatening women's life and health [1]. Numerous studies have reported mortality changes in the three primary reproductive system cancers among different countries, with inconsistent temporal trends. Because of the large population base and severe aging, the number of deaths in China accounts for 17.51% of the world on malignant tumors of the female reproductive system [2].

Studies have shown that the mortality rate of cervical cancer and ovarian cancer in China has increased to varying degrees, becoming a stark public health issue [3, 4]. In recent years, patients with cervical cancer are younger, tend to present at an early stage [5]. The openness of sexual attitudes (such as premature sex life and multiple sexual partners) increased the risk of human papillomavirus (HPV) infection in the adolescent population, which is an important reason for the younger trend of cervical cancer [6]. The expansion of screening and the improvement of diagnostic capabilities provided a technical support for detecting more potential malignant tumors of the female reproductive system [7]. In the context of the prevalence of risk factors, the mortality and disease burden caused by malignant tumors of the female reproductive system needs to be further analyzed.

Since 2004, the Chinese center for disease control and prevention has conducted all cause surveillance of death in 31 provinces (excluding Hong Kong, Macau, and Taiwan) every year, covering 6–24% of the national population, which represents the Chinese population [8]. This study aims to evaluate trends of cervical cancer, uterine cancer, and ovarian cancer mortality and disease burden from 2006 to 2020 across age groups and periods. It will help optimize existing screening guidelines, achieve accurate early screening and warning of high-risk groups, and improve women's health.

## Materials and methods

### Data sources

The latest death data of malignant tumors of the female reproductive system in China were obtained from the "China death cause surveillance dataset" from 2006 to 2020. An additional file shows this in more detail (see Additional file 1). The content includes the number of women in each age group in the monitoring area and the number of deaths. These tumors were coded according to the International Classification of Diseases, Tenth Revision, mainly including cervical cancer, uterine cancer and ovarian cancer, and the codes were C53, C54 and C56, respectively. Patients with non-primary tumors and unknown cause of death were excluded.

### Statistical techniques

The Joinpoint 4.9.1.0 software was used to calculate crude mortality rate (CMR), age-standardized mortality rate (ASMR), annual percentage change (APC), and average APC (AAPC) to evaluate the trend of mortality of the three types of malignancies. CMR was used to compare the mortality of different diseases in the surveillance population in the same year, and ASMR was used to compare the mortality of conditions in different years. APC was calculated based on log-linear regression. The model formula is: log(ASMRy) = α + βy (y is the year, α is a constant, and β is the regression coefficient), then APC = {exp(β_j_ − 1)} × 100% (j index the segments in the desired range of years) [9]. AAPC is the weighted average of the APCs of multiple trend stages according to the number of years, then AAPC = {exp(∑w_j_β_j_/∑w_j_) − 1} × 100% (w_j_ as the length of each segment in the range of years). If the 95% confidence interval (*CI*) of AAPC contains zero, then there is no evidence to reject the null hypothesis that the change has no statistical significance. Otherwise, it has statistical significance if 95%*CI* of AAPC is different from zero. Up to 3 connection points could be set in the monitoring data of 15 years, and the optimal model was selected based on permutation test.

Loss of life expectancy (LLE), years of life lost (YLL), and YLL rates were used as indicators of disease burden and calculated through the life table and disease burden table [10]. The LLE calculation formula is: $\Delta {\text{e}}_{0} = {\text{e}}_{0}^{\prime } - {\text{e}}_{0}$(${\text{e}}_{0}^{\prime }$ is the life expectancy at birth of a woman without the disease, and e^0^ is the life expectancy at birth with the disease). YLL was obtained by multiplying the number of deaths by age group by the corresponding standard life expectancy [11]. YLL rate is the ratio of YLL to the number of monitored populations. The female population data of China's sixth census in 2010 was selected as the standard population. The inspection level was set to α = 0.05.

## Results

### Mortality of malignant tumors of the female reproductive system

**Fig. 1:**
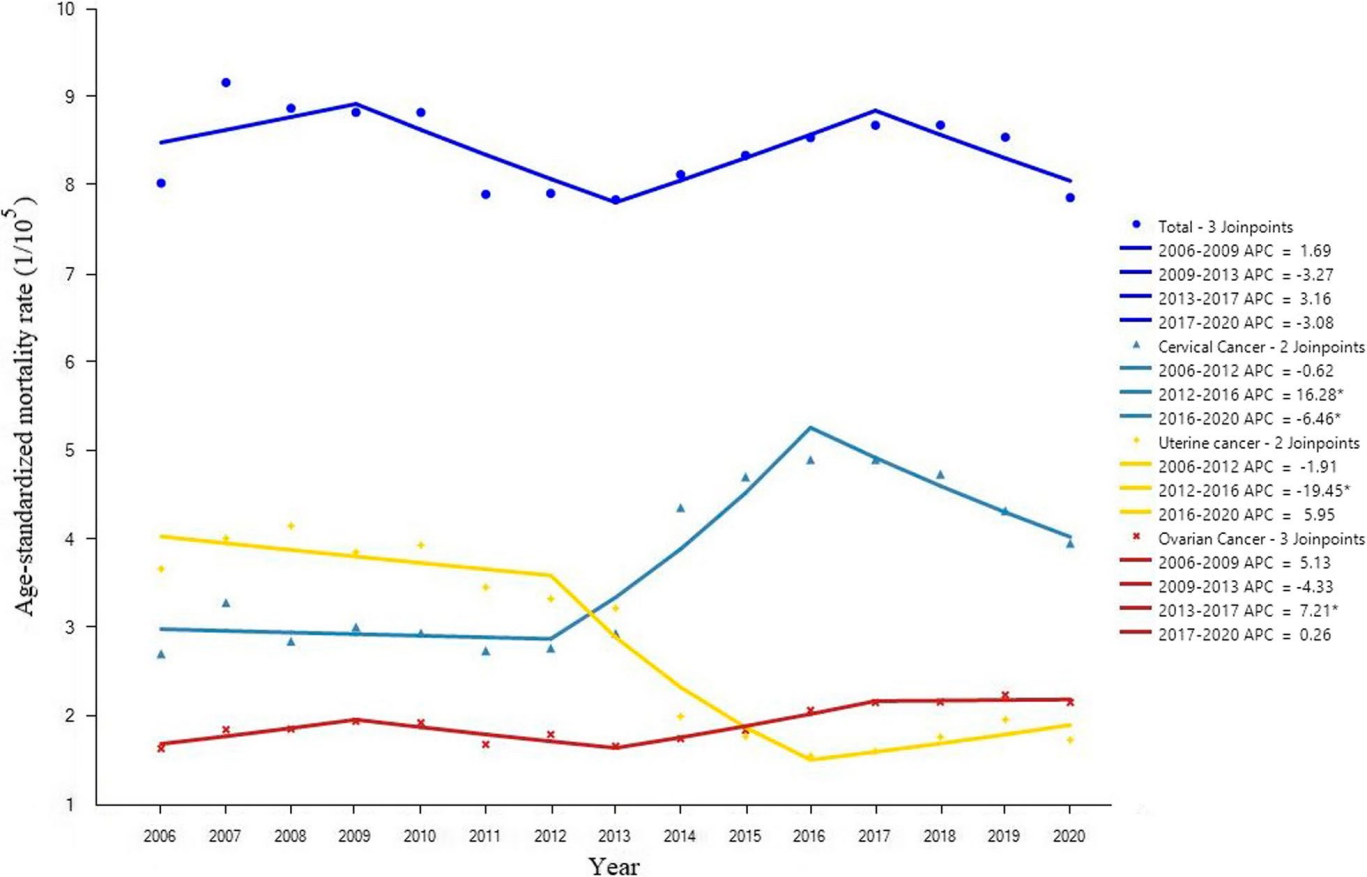
Trend of mortality of malignant tumors of the female reproductive system in China, 2006–2020

From 2006 to 2020, the overall CMR of malignant tumors of the female reproductive system in the national surveillance areas was between 7.0/10^5^ and 100.77/10^5^, and the ASMR showed no significant change (Table 1). From 2006 to 2013, the CMR of uterine cancer was higher than that of cervical cancer and ovarian cancer. While from 2014 to 2020, the CMR of cervical cancer was higher than that of ovarian cancer and uterine cancer. The ASMR of cervical cancer showed an upward trend from 2012 to 2016 (APC = 16.28%) and a downward trend from 2016 to 2020 (APC =  − 6.46%). Still, the overall change trend during the monitoring period was not statistically significant (*P* > 0.05) (Fig. 1). The ASMR of uterine cancer showed a downward trend from 2006 to 2020 (AAPC =  − 5.21%, 95%*CI*: − 9.31% ~  − 0.91%). The ASMR of ovarian cancer showed an upward trend from 2013 to 2017 (APC = 7.21%), but the overall change from 2006 to 2020 was not statistically significant.

**Table 1:** Changes in mortality of malignant tumors of the female reproductive system in China (1/10^5^)

Year	Cervical cancer		Uterine cancer		Ovarian cancer		Total	
	CMR	ASMR	CMR	ASMR	CMR	ASMR	CMR	ASMR
2006	2.37	2.72	3.21	3.68	1.42	1.64	7.00	8.04
2007	2.94	3.29	3.56	4.02	1.65	1.86	8.14	9.17
2008	2.66	2.86	3.83	4.16	1.72	1.87	8.21	8.88
2009	2.90	3.02	3.73	3.86	1.86	1.95	8.49	8.84
2010	2.89	2.95	3.84	3.95	1.91	1.94	8.64	8.83
2011	2.85	2.75	3.58	3.47	1.78	1.69	8.21	7.91
2012	2.95	2.78	3.56	3.34	1.92	1.80	8.43	7.92
2013	3.27	2.95	3.61	3.23	1.87	1.67	8.76	7.85
2014	4.91	4.37	2.28	2.01	1.98	1.76	9.17	8.13
2015	5.30	4.72	2.01	1.78	2.08	1.85	9.40	8.35
2016	5.65	4.91	1.83	1.56	2.41	2.08	9.89	8.55
2017	5.68	4.91	1.88	1.61	2.53	2.16	10.09	8.69
2018	5.58	4.75	2.14	1.78	2.58	2.17	10.30	8.69
2019	5.39	4.33	2.52	1.97	2.86	2.25	10.77	8.55
2020	5.14	3.96	2.33	1.74	2.87	2.17	10.35	7.87
AAPC	4.86	2.16	− 2.21	− 5.21	5.03	1.87	2.54	− 0.37
95% CI	1.33,8.51	− 1.67,6.13	− 6.23,1.98	− 9.31, − 0.91	0.99,9.22	− 1.7,5.57	2.07,3.00	− 3.07,2.4
*t*	2.72	1.10	− 1.05	− 2.36	2.45	1.02	11.92	− 0.27
*P*	0.007	0.273	0.296	0.018	0.014	0.309	< 0.001	0.789

*AAPC* Average annual percentage change; *ASMR* Age-standardized mortality rate; *CMR* Crude mortality rate

### Age-specific mortality of malignant tumors of the female reproductive system

**Fig. 2:**
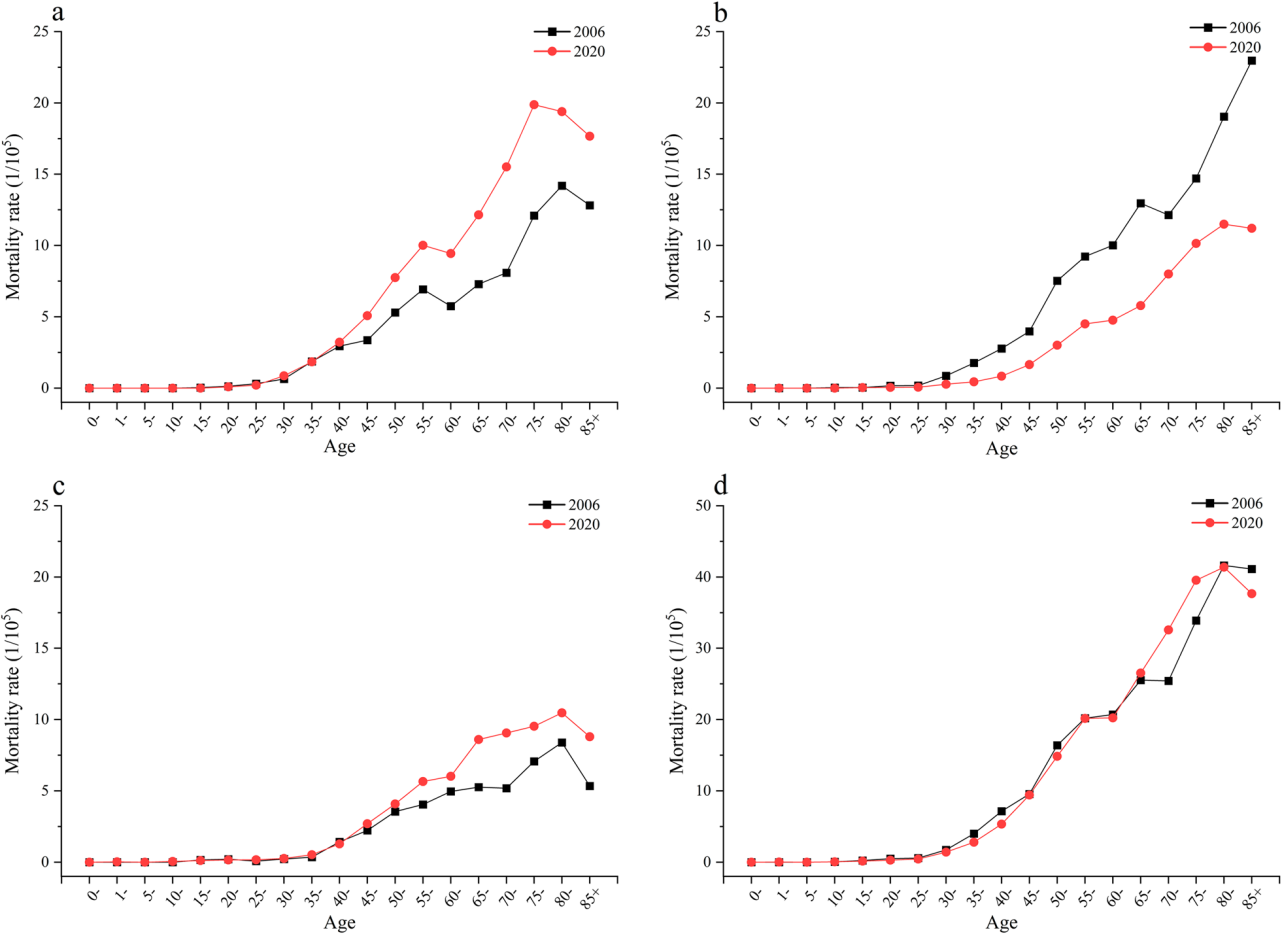
The mortality changes of malignant tumors of the female reproductive system by age. **a** Cervical cancer, **b** Uterine cancer, **c** Ovarian cancer, **d** Total

In terms of age, the mortality rate of Chinese women from malignant tumors of the reproductive system is low before 30 years old (Fig. 2). However, it increases rapidly with age after 30 years old, especially over 65. In 2020, the mortality of uterine cancer in women decreased in the age group over 30 years old, while the mortality of cervical cancer and ovarian cancer increased in the age group over 45.

### Disease burden of malignant tumors of the female reproductive system

During the study period, the overall YLL rate of malignant tumors of the female reproductive system in China ranged from 90.87/10^5^ to 123.39/10^5^, and there was no statistically significant change in the standardized YLL rate (AAPC =  − 1.09%, 95%*CI*: − 2.48% ~ 0.32%) (Table 2). The standardized YLL rate of uterine cancer showed a downward trend (AAPC =  − 6.07%, 95%*CI*: − 9.45% ~  − 2.58%), while the standardized YLL rate for cervical cancer and ovarian cancer did not change significantly. In 2006, the YLL rate of uterine cancer was higher than that of cervical and ovarian cancer, and the YLL rate of cervical cancer was higher than that of ovarian and uterine cancer in 2020.

**Table 2:** YLL rate of malignant tumors of the female reproductive system in China (1/10^5^)

Year	Cervical cancer		Uterine cancer		Ovarian cancer		Total	
	YLL rate	Standardized YLL rate	YLL rate	Standardized YLL rate	YLL rate	Standardized YLL rate	YLL rate	Standardized YLL rate
2006	32.13	36.19	40.43	45.55	18.31	20.95	90.87	102.69
2007	39.46	43.88	46.16	51.65	20.31	22.80	105.93	118.33
2008	33.84	36.04	47.00	50.58	21.97	23.59	102.81	110.21
2009	37.55	38.12	47.61	48.02	22.32	22.76	107.48	108.90
2010	37.88	37.58	46.31	45.95	23.20	22.92	107.39	106.45
2011	38.98	37.63	44.01	42.19	21.88	20.88	104.87	100.70
2012	40.30	38.47	42.90	40.72	23.44	22.31	106.64	101.49
2013	42.82	39.32	43.66	39.70	22.33	20.19	108.81	99.21
2014	62.89	57.51	26.27	23.67	24.8	22.64	113.96	103.82
2015	66.06	60.45	23.39	21.31	24.42	22.32	113.87	104.08
2016	70.90	65.02	20.53	18.44	28.8	26.15	120.23	109.62
2017	69.94	63.60	21.75	19.57	29.63	26.61	121.32	109.78
2018	67.59	61.75	23.13	20.65	29.86	26.91	120.58	109.31
2019	64.08	54.76	26.71	22.04	32.60	27.27	123.39	104.06
2020	60.02	50.04	24.38	19.48	32.58	26.50	116.98	96.02
AAPC	4.20	1.99	− 3.60	− 6.07	4.20	1.03	1.51	− 1.09
95% CI	0.27,8.29	− 2.02,6.16	− 7.67, − 0.66	− 9.45, − 2.58	3.36,5.05	− 1.05,3.15	1.01,2.01	− 2.48,0.32
*t*	2.10	0.96	− 1.66	− 3.36	10.97	0.96	6.59	− 1.52
*P*	0.036	0.335	0.097	< 0.001	< 0.001	0.335	< 0.001	0.128

*AAPC* Average annual percentage change; *YLL* Years of life lost

### Age-specific disease burden of malignant tumors of the female reproductive system

**Fig. 3:**
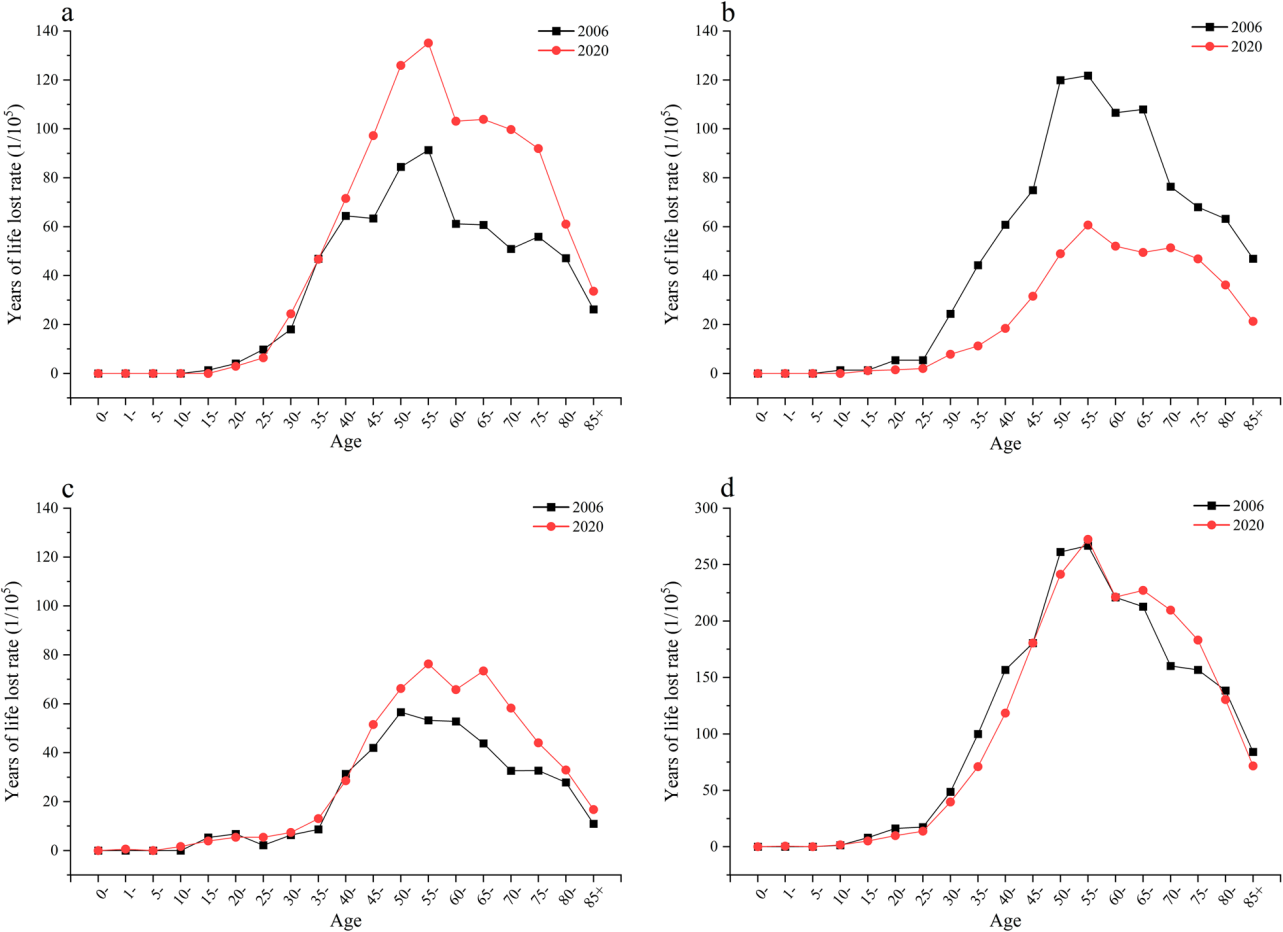
Years of life lost rate changes of malignant tumors of the female reproductive system by age. **a** Cervical cancer, **b** Uterine cancer, **c** Ovarian cancer, **d** Total

**Fig. 4:**
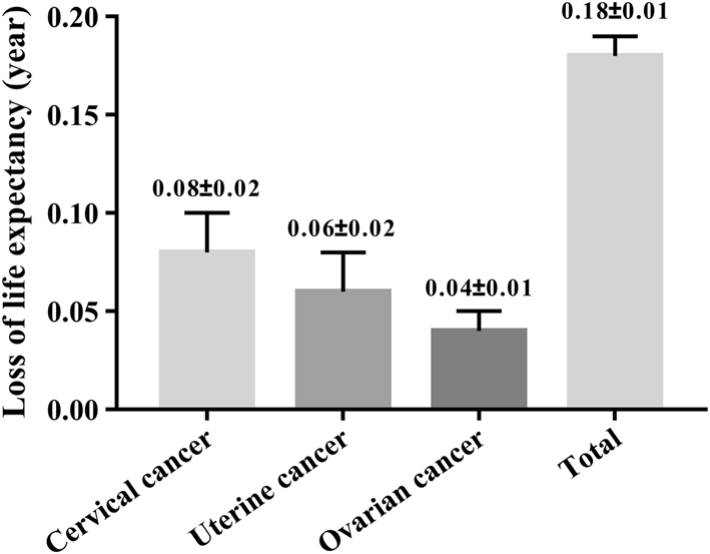
Loss of life expectancy among Chinese women due to malignant tumors of the female reproductive system

The overall YLL rate of malignant tumors of the female reproductive system in China increased rapidly at 30–54, reached the highest at 55–59, and then decreased (Fig. 3). In 2020, the YLL rate of uterine cancer in women of all age groups had decreased significantly compared with that in 2006, while the YLL rates of cervical cancer and ovarian cancer have increased significantly at 45–79. From 2006 to 2020, the average LLE at birth because of cervical cancer, uterine cancer, ovarian cancer, and total deaths among Chinese women was 0.08, 0.06, 0.04, and 0.18 years, respectively (Fig. 4).

## Discussion

In 2020, about 247,000 women in China suffered from malignant tumors of the reproductive system, and 113,186 died, posing a severe threat to women's life and health [2]. This study analyzed the mortality, YLL and LLE of cervical cancer, uterine cancer and ovarian cancer using the National Death Cause Surveillance Dataset from 2006 to 2020,, which fully reflect the severity of the three cancers. Joinpoint regression model was used to analyze the long-term trend of the burden of the three cancers, which is scientific segmentation and good fitting. The different trends of mortality and disease burden among the three types of cancer may be the combined effects of screening, improved diagnosis and treatment techniques, increased exposure to risk factors, and changes in reproductive characteristics [12].

### Cervical cancer

About 59,000 women died of cervical cancer in China in 2020, accounting for 17.28% of the global total. This study found that cervical cancer ASMR showed an upward trend from 2013 to 2017 and a downward trend from 2017 to 2020, consistent with the observed trends worldwide [7]. The YLL rate of cervical cancer increased significantly in women aged 45–79, suggesting that middle-aged and older adults are the key population in reducing the disease burden. Cervical cancer is a preventable tumor, and studies have determined the effectiveness of the HPV vaccine and the importance of early vaccination [13]. In addition, some specific biomarkers (such as peritoneal HPV-DNA, CEA, SCC-Ag, CD44) are helpful to identify early cervical cancer, make the best treatment plan for patients and improve the prognosis [14, 15]. The United States has adopted effective screening, prevention and control, and the incidence and mortality of cervical cancer in women have decreased significantly [16]. The World Health Organization (WHO) launched the "Global strategy to accelerate the elimination of cervical cancer" in November 2020 [17]. Vaccination, screening and treatment are the key measures. The first Chinese domestic HPV vaccine "Cecolin" was pre-qualified by WHO in 2021. Increasing production, reducing prices, and promoting the inclusion of vaccines in national immunization programs are effective ways to promote HPV vaccination [18]. Since 2019, China has included cervical cancer screening in essential public health services to improve coverage. Liquid-based thin-layer cytology and HPV testing are the primary measures for cervical cancer screening, which are highly accurate. According to the "China national program for women's development (2021–2030)" formulated by the Chinese government, more than 70% of women will complete cervical cancer screening by 2030, providing policy support for the international strategic goal of "90–70-90" [19].

### Uterine cancer

The overall incidence of uterine cancer is on the rise worldwide, especially in developed countries [20]. This study shows that before 2013, uterine cancer was also the leading cause of death in malignant tumors of the female reproductive system in China. Mortality and disease burden of uterine cancer decreased significantly in women of all age groups between 2006 and 2020. Advances in surgery, radiotherapy and chemotherapy have improved the prognosis of patients with uterine cancer, with a higher survival rate than other gynecological cancers [21]. Uterine cancer mainly occurs in postmenopausal women, and long menstrual periods and low parity increase the risk, which is related to changes in estrogen and progesterone [22]. The presence of obesity and diabetes, which increases circulating levels of estrogen, are two important risk factors for uterine cancer [23]. In addition, evidence indicates peroxisome proliferator-activated receptors (PPARs) play a key role in obesity, metabolic diseases and cancer by regulating cell proliferation and differentiations [24]. The increased risk of sentinel lymph node mapping failure in patients with apparent early-stage endometrial cancer is also associated with obesity [25]. Endometrial cancer is the primary pathological type of uterine cancer and is the most common gynecological malignancy in high-income countries, while uterine sarcoma is rare [26]. Currently, there is no effective population screening method for uterine cancer. The first symptom is uterine bleeding, which could be combined with aging and genetic susceptibility as the basis for screening key populations [27]. Health management and monitoring of high-risk groups of uterine cancer should be implemented to ensure timely diagnosis and treatment.

### Ovarian cancer

Ovarian cancer has the highest case fatality rate among gynecological malignancies, with an estimated 55,000 new cases and 38,000 deaths in China in 2020 [2]. Although the ASMR of ovarian cancer in Chinese women increased from 2013 to 2017, there was no obvious change during the study period. The prevalence of risk factors, such as obesity, smoking, and menopausal hormone therapy, has increased the prevalence of ovarian cancer. In contrast, childbirth and breastfeeding are highly protective against the development of ovarian cancer [28]. Oral contraceptives have the effect of inhibiting ovulation, controlling luteinizing hormone, and reducing follicle-stimulating hormone. In the early 1960s, oral contraceptives became widely available in Europe, and the incidence of ovarian cancer in women decreased significantly [29]. Ovarian cancer mainly occurs in women after age 35, with the highest prevalence at 55–59. The effect of ovarian cancer treatment and prognosis is still poor, and the mortality and YLL rate in women over 45 increased from 2006 to 2020. Therefore, an accurate screening model of ovarian cancer risk groups should be established as soon as possible and carry a tertiary prevention strategy. Kobayashi Y et al. evaluated various prognostic models of ovarian cancer based on clinical indicators and molecular markers to provide a scientific basis for guiding patients' treatment decisions, which need to be validated in the Chinese female population [30].

However, some limitations in this study should be considered. We have not analyze the disease burden of vulvar cancer and uterine sarcoma due to unavailable data in the National Death Surveillance Database. Due to the lack of morbidity information, Disability Adjusted Life Year (DALY) and Years Lived with Disability (YLD) as the other two indicators of disease burden were not calculated because the surveillance content was mainly death data. Additionally, the latest available surveillance data is currently 2020.

## Conclusions

From 2006 to 2020, the mortality rate and disease burden of uterine cancer in Chinese females decreased, and that of cervical cancer and ovarian cancer was still high. Aging, obesity, diabetes, hormone replacement therapy, and an unhealthy lifestyle are risk factors for the three types of cancer. Malignant tumors of the female reproductive system are closely related to the national fertility rate and are the fundamental guarantee for the normal development of the population. Medical resources should be distributed fairly, and it should improve tertiary prevention to protect women's health.

## Supplementary Information

**Additional file 1**: The data that support the findings of this study. Cancer: three types of malignant tumors of the female reproductive system, Year: monitoring year of 2006–2020, Age group: 19 age groups were divided, Count: the number of deaths, Population: the number of people monitored, Standard population: the age distribution of females in the sixth census of China in 2010.

## Data Availability

The datasets analyzed during the current study are available in the China Death Surveillance Database (https://ncncd.chinacdc.cn/xzzq_1).

## Abbreviations

ASMR: Age-standardized mortality rate
APC: Annual percentage change
AAPC: Average annual percentage change
CMR: Crude mortality rate
CI: Confidence interval
DALY: Disability adjusted life year
GLOBOCAN: Global Cancer Statistics
HPV: Human papillomavirus
ICD: International Classification of Diseases
LLE: Loss of life expectancy
WHO: World Health Organization
YLD: Years lived with disability
YLL: Years of life lost
